# Development of an HPLC-MS/MS Method for Chiral Separation and Quantitation of (*R*)- and (*S*)-Salbutamol and Their Sulfoconjugated Metabolites in Urine to Investigate Stereoselective Sulfonation

**DOI:** 10.3390/molecules28207206

**Published:** 2023-10-21

**Authors:** Lukas Corbinian Harps, Annika Lisa Jendretzki, Clemens Alexander Wolf, Ulrich Girreser, Gerhard Wolber, Maria Kristina Parr

**Affiliations:** 1 Pharmaceutical Analysis, Institute of Pharmacy, Freie Universität Berlin, Königin-Luise-Straße 2+4, 14195 Berlin, Germany; lukas.harps@fu-berlin.de (L.C.H.); annika.jendretzki@fu-berlin.de (A.L.J.); 2 Pharmaceutical and Medicinal Chemistry (Computer-Aided Drug Design), Institute of Pharmacy, Freie Universität Berlin, Königin-Luise-Straße 2+4, 14195 Berlin, Germany; ca.wolf@fu-berlin.de (C.A.W.); gerhard.wolber@fu-berlin.de (G.W.); 3 Institute of Pharmacy, Christian-Albrechts University Kiel, Gutenbergstr. 76, 24118 Kiel, Germany; girreser@pharmazie.uni-kiel.de

**Keywords:** enzymatic stereoselectivity, molecular docking, bioanalysis, quality-by-design

## Abstract

The aim of this study was to develop and optimize a chiral HPLC-MS/MS method for quantitative analysis of (*R*)-/(*S*)-salbutamol and (*R*)-/(*S*)-salbutamol-4′-*O*-sulfate in human urine to allow for bioanalytical quantitation of the targeted analytes and investigations of stereoselectivity in the sulfonation pathway of human phase Ⅱ metabolism. For analytical method development, a systematic screening of columns and mobile phases to develop a separation via enantiomerically selective high performance liquid chromatography was performed. Electrospray ionization settings were optimized via multiple-step screening and a full factorial design-of-experiment. Both approaches were performed matrix-assisted and the predicted values were compared. The full factorial design was superior in terms of prediction power and knowledge generation. Performing a longitudinal excretion study in one healthy volunteer allowed for the calculation of excretion rates for all four targeted analytes. For this proof-of-concept, either racemic salbutamol or enantiopure levosalbutamol was administered perorally or via inhalation, respectively. A strong preference for sulfonation of (*R*)-salbutamol for inhalation and peroral application was found in *in vivo* experiments. In previous studies phenol sulfotransferase 1A3 was described to be mainly responsible for salbutamol sulfonation in humans. Thus, in vitro and in silico investigations of the stereoselectivity of sulfotransferase 1A3 complemented the study and confirmed these findings.

## 1. Introduction

Salbutamol is a short-acting beta-2 sympathomimetic drug which is widely used for the acute treatment of bronchial asthma and chronic obstructive pulmonary disease [1,2,3,4,5]. In sports, the treatment of athletes and therefore the application via inhalation of the racemic drug in and out of competition is allowed, according to the regulations of the World Anti-Doping Agency (WADA prohibited list [6]), but limited to doses of 600 µg/8 h and 1600 µg/24 h and a threshold of 1000 ng/mL (decision limit 1200 ng/mL) of the racemic drug in urinary doping control analysis. Administration of the pure, pharmacologically active enantiomer levosalbutamol is prohibited in and out of competition at all times, independent of dose or administration route.

The metabolism of salbutamol in humans is known to mainly result in sulfonation via the phenol sulfotransferase 1A3 (SULT1A3) to the inactive salbutamol-4′-*O*-sulfate. Glucuronidation is of minor importance and was reported to be up to only 3% [7]. Studies reported a non-activity towards salbutamol for several other phenol SULTs such as SULT1A1, SULT1B1, and SULT1C4. The isoform SULT1A3 is mainly located in the small intestine but also in the liver [8,9,10,11]. A stereoselective transformation in favor of active levosalbutamol was reported by Boulton et al. [9] and Walle et al. [12]. Furthermore, (*S*)-salbutamol has been reported to inhibit the enzymatic sulfonation of (*R*)-salbutamol, resulting in reduced elimination and thus a higher concentration of levosalbutamol in plasma when applied as racemate [13].

An approach to identifying the administration of pure levosalbutamol via achiral chromatography from observation of the proportion of the sulfonated metabolite was reported by Jendretzki et al. [14]. Additionally, peroral administration and inhalation were identified in the same manner.

While chiral separation and quantitation of (*R*)-, and (*S*)-salbutamol have been performed and published in several studies [11,15,16,17,18,19] quantitation of their sulfoconjugated metabolites has rarely been performed and published. The bottleneck was the availability of the reference substances. However, most popular for chiral separation of salbutamol enantiomers in reversed-phase high-performance liquid chromatography (HPLC) are teicoplanin-based columns [11,15,16] which also enable chiral separation of the sulfoconjugates [20].

Joyce et al. [20] performed simultaneous chiral separation of salbutamol and 4′-*O*-metabolites in human urine and plasma samples and thus published a validated method for quantitation. Ward et al. [21] adapted the method and performed an excretion study in which racemic salbutamol was administered via inhalation (1200 µg), perorally (2 mg), or intravenously (500 µg). Concentrations in plasma and urine were monitored, and a comprehensive picture of the pharmacokinetics of (*R*)-salbutamol, (*S*)-salbutamol, (*R*)-salbutamol-4′-*O*-sulfate, and (*S*)-salbutamol-4′-*O*-sulfate was presented. The results showed strong chiral shifts in urinary excretion and in plasma samples which were also dependent on the intake route. Five years later (2005), the FDA approved pure levosalbutamol as drug for the treatment of asthma patients. To the best of our knowledge, no reports on the sulfoconjugate quantitation of levosalbutamol have been published so far. Thus, this study provides additional data that will improve understanding of stereoselective salbutamol sulfonation.

## 2. Results

### 2.1. HPLC-MS/MS Method Development

#### 2.1.1. Chiral Chromatography

**Figure 1:**
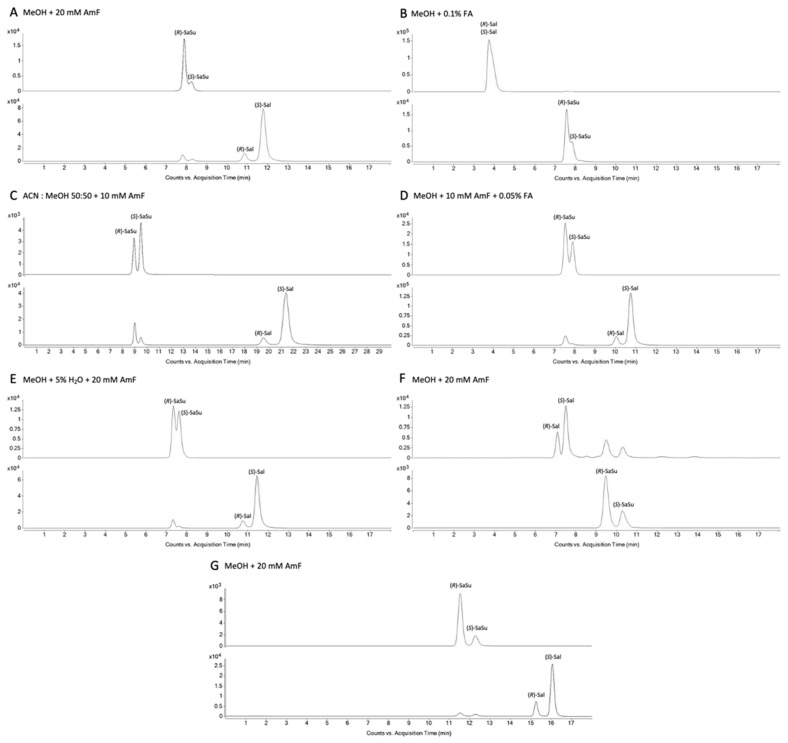
Chromatograms from method development for the most promising stationary phases teicoplanin and vancomycin. A teicoplanin column was chosen for the testing of mobile phase compositions. (**A**–**E**) Chromatograms using a teicoplanin-based column (4.6 mm I.D. × 100 mm; 2.7 µm, poroshell) for different mobile phase compositions and (**F**) for comparison with a vancomycin-based column (4.6 mm I.D. × 100 mm; 2.7 µm, poroshell). Chromatograms obtained with the finally optimized method are shown in (**G**) using a teicoplanin-based column (4.6 mm I.D. × 150 mm; 2.7 µm, poroshell).

**Figure A1:**
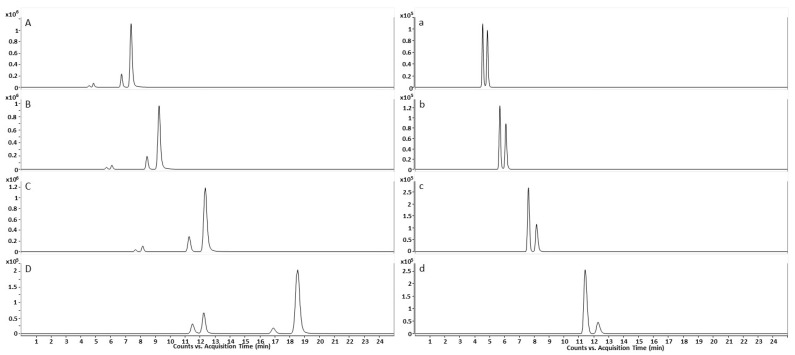
Chromatograms on the left (**A**–**D**) show salbutamol ([M + H]^+^ = 240.0 → 148.1); chromatograms on the right (**a**–**d**) show salbutamol-4′-*O*-sulfate ([M − H]^−^ = 318.0 → 218.1). Flow rates were 0.5 mL/min (**A**,**a**), 0.4 mL/min (**B**,**b**), 0.3 (**C**,**c**), and 0,2 mL/min (**D**,**d**). Separation of an excretion sample was conducted on a teicoplanin-based column (4.6 mm I.D. × 150 mm, 2.7 µm, solid core particles) at 35 °C, mobile phase was 20 mM AmF in methanol, and isocratic elution mode was applied.

**Figure A2:**
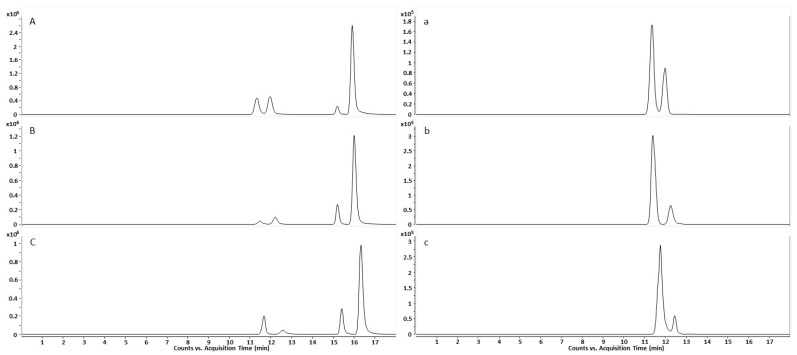
Chromatograms on the left (**A**–**C**) show salbutamol ([M + H]^+^ = 240.0 → 148.1); chromatograms on the right (**a**–**c**) show salbutamol-4′-*O*-sulfate ([M − H]^−^ = 318.0 → 218.1). The final method was applied with column temperature at 35 °C (**B**,**b**). Chromatogram resulting from column temperature of 50 °C (**A**,**a**) and 20 °C (**C**,**c**). Separation of an excretion sample was conducted on a teicoplanin-based column (4.6 mm I.D. × 150 mm, 2.7 µm, solid core particles), mobile phase was 20 mM AmF in methanol, and isocratic elution mode was applied at flow rates 0.2 mL/min for 13.55 min and was then increased to 0.4 mL/min.

The column and the mobile phase screening developed via one-factor-at-a-time (OFAT) design eliminated the stationary phases based on derivatized cyclofructan and hydroxypropylated-β-cyclodextrin due to insufficient selectivity for enantiomer separation. Vancomycin (Figure 1F) and teicoplanin (Figure 1A) showed selectivity towards the four analytes (*R*)-salbutamol ((*R*)-Sal), (*S*)-salbutamol ((*S*)-Sal), (*R*)-salbutamol-4′-*O*-sulfate ((*R*)-SaSu), and (*S*)-salbutamol-4′-*O*-sulfate ((*S*)-SaSu). The teicoplanin-based column gave promising results in terms of selectivity and peak shape. Evaluation of several mobile phase compositions led to the separation of the analytes (Figure 1). When the observed effects of mobile phase changes were compared to the separation using methanol (MeOH) with 20 mM ammonium formate (AmF) the addition of 50% acetonitrile (ACN) (Figure 1C) increased retention time and thereby resolution. The combination of 15 mM ammonium formate and 0.025% formic acid (FA) also resulted in good separation, whereas combinations with higher amounts of formic acid and less ammonium formate impaired the chiral selectivity (Figure 1B,D). Furthermore, only low amounts of water were tolerated, and the more water was added to the mobile phase, the more the chiral separation was compromised (Figure 1E). To allow for robust quantitation over a high range of analyte concentrations and therefore peak height and width, a 150 mm-long teicoplanin-based column was chosen for further optimization. A re-evaluation of most screening parameters was performed. Additionally changes in temperature and flow rate were investigated, and several experiments on gradients including at least small amounts of water were performed. The chromatographic behavior of salbutamol and salbutamol-4′-*O*-sulfate as a function of the flow rate is shown in Figure A1. In Figure A2, changes in separation and peak shape using the final method at higher or lower column temperature are presented.

A very good resolution with a mean value of *R_s,Sal_* = 2.88 (minimum resolution 2.59, maximum 3.48 throughout all sample concentrations) for (*R*)- and (*S*)-salbutamol and *R_s,SaSu_* = 2.08 (minimum resolution 1.65, maximum 2.72 throughout all sample concentrations) for (*R*)- and (*S*)-salbutamol-4′-*O*-sulfate within an acceptable run time were found using an uncommon isocratic approach using MeOH with 20 mM ammonium formate applying two different flow rates in the chromatographic run (Figure 1G).

Retention time stability was monitored throughout all runs. The mean values of the retention time were 11.53 min for (*R*)- and 12.31 min for (*S*)-salbutamol-4′-*O*-sulfate with maximum deviations of 0.05 min and 0.07 min, respectively. Furthermore, (*R*)-salbutamol was eluted at 15.25 min and (*S*)-salbutamol at 16.05 min with maximum deviations of 0.03 min for (*R*)- and 0.04 min for (*S*)-salbutamol. The deviation in retention time did not exceed the tolerance of 1% at any time.

#### 2.1.2. Electrospray Ionization

##### Multiple-Step Screening Design of Experiment (MSSD)

**Figure 2:**
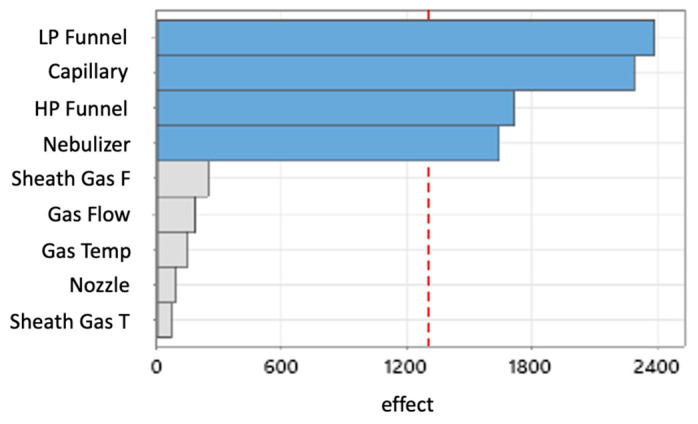
Pareto diagram of the effects (effect) of low-pressure funnel voltage (LP Funnel), capillary voltage (Capillary), high-pressure funnel voltage (HP funnel), nebulizer pressure (Nebulizer), sheath gas flow (Sheath Gas F), drying gas flow (Gas Flow), drying gas temperature (Gas Temp), nozzle voltage (Nozzle), and sheath gas temperature (Sheath Gas T) on the abundance of the quantifier transition (*m*/*z* = 318.0 → 218.1) of salbutamol-4′-*O*-sulfate. The analyte was injected from a urine sample and ionized in ESI− mode. Red dashed line is the reference line for significance. Blue bars that cross the reference line highlight parameters that have a significant effect on the abundance of the quantifier transition, grey bars indicate there is no significant effect.

**Figure A3:**
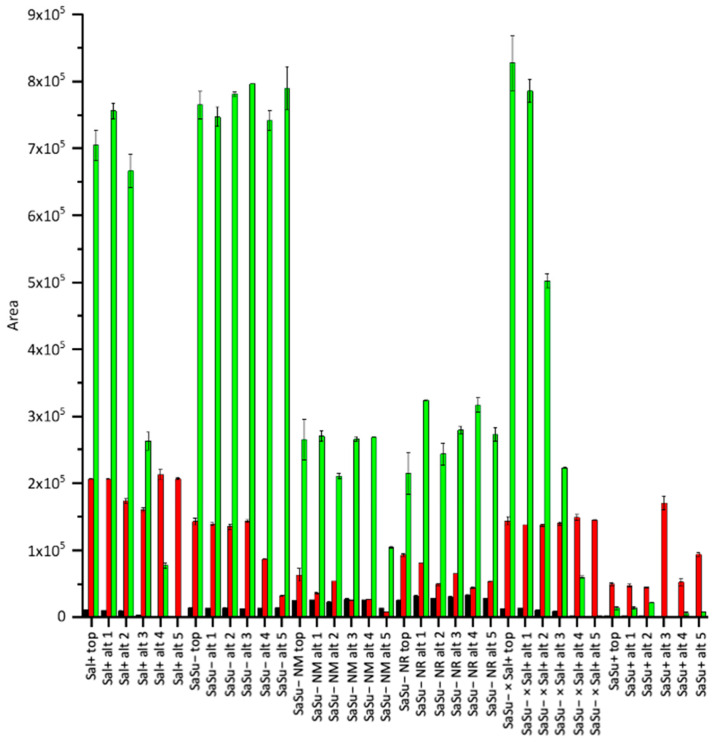
Results of the verification runs for the predicted optimal ionization conditions (top) and the five suggested alternative conditions (alt 1–5). Green bars show salbutamol (Sal) [M + H]^+^ = 240.0 → 148.0, red (SaSu−) and black bars (SaSu+) show salbutamol-4′-*O*-sulfate. The multiple-step screening experiments for the quantifiers of salbutamol (Sal) and salbutamol-4′-*O*-sulfate (SaSu) were performed in MRM for ESI− (−) or ESI+ (+) mode. The analytes were injected from a spiked urine sample (matrix-assisted). No matrices (NM) were used for the multiple-step screening. More narrow ranges (NRs) for the nine factors were used. Four factors were independently adjustable for ESI− and ESI+ mode (capillary voltage, nozzle voltage, and low- and high-pressure funnel voltage). For these, multiple-step screenings were performed for optimization of sal in ESI+ while keeping the predicted optimum for SaSu in ESI− (SaSu− × Sal+). When optimization for salbutamol in ESI+ (Sal+) was performed, the before optimized conditions for SaSu in ESI− were applied to capillary voltage, nozzle voltage, and low- and high-pressure funnel voltage.

In the optimization of the ion source parameters for improved sensitivity, a multiple-step screening design of experiment (MSSD) was chosen. The multiple-step screening approach identified in the first screening low-pressure funnel voltage, capillary voltage, high-pressure funnel voltage, and nebulizer pressure as the variables with significant impact on the abundance of the salbutamol-4′-*O*-sulfate quantifier (Figure 2; Figure A3: SaSu−). The factors with minor impact were kept constant.

**Figure 3:**
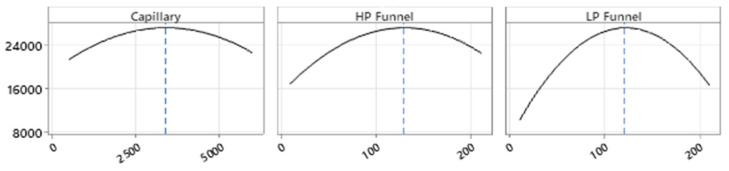
Quadratic model for the impact on the ion abundance (Y-axes) of the quantifier of salbutamol-4′-*O*-sulfate in MRM mode (*m*/*z* = 318.0 → 218.1). The predicted optima are marked with vertical dashed blue lines. For capillary 3400 V, for HP funnel 129 V, and for LP funnel, 121 V were predicted as optima. *X*-axes show voltages for capillary, high-pressure funnel, or low-pressure funnel.

In the next two steps, the fit of a linear response model was tested and a curvature was detected. Thus, the linear model was adjusted to a quadratic model. Here, the influence of the nebulizer pressure was identified to be statistically non-significant and therefore was taken out from the prediction model (Figure 3).

One top prediction and four alternative predictions were provided and subsequently compared with experimental data. The top prediction was assessed as superior to the alternatives. Based on this, further experiments were performed to find optimized ionization conditions for the quantifier of salbutamol-4′-*O*-sulfate (*m*/*z* = 320.0 → 220.0) and salbutamol (*m*/*z* = 240.0 → 148.0) in ESI+ (Figure A3: SaSu+ and Sal+). The ion source settings allow for the use of different values for capillary voltage, nozzle voltage, high-pressure and low-pressure funnel voltage when operated in ESI+ and ESI− in one method. The set values for nebulizer pressure, drying gas temperature, drying gas flow, sheath gas, and sheath gas temperature apply for both modes, ESI+ and ESI−. Salbutamol was analyzed in ESI+ and salbutamol-4′-*O*-sulfate was analyzed in ESI−. In a further step, the optimized ESI− method for salbutamol-4′-*O*-sulfate was adjusted in terms of capillary voltage, nozzle voltage and high-pressure and low-pressure funnel voltage to account for the quantifier of salbutamol (*m*/*z* = 240.0 → 148.0) in ESI+ (Figure A3: SaSu− × Sal+). Furthermore, the screening was performed in ESI− mode for salbutamol-4′-*O*-sulfate injected from neat methanol, and the predicted conditions were evaluated and compared to the matrix-assisted optimization (Figure A3: SaSu− NM). The results are presented in the Appendix A (Figure A3). The highest abundance for the quantifier of salbutamol-4′-*O*-sulfate was generated by the ionization factor settings found in the screening process for the optimization of salbutamol. Notably, the before predicted values (SaSu, Figure A3, top) for capillary voltage, nozzle voltage, and low- and high-pressure funnel voltage in ESI− were kept.

The achieved data showed highest area for salbutamol-4′-*O*-sulfate (*m*/*z* = 318.0 → 218.1) in the top prediction and the five alternatives for salbutamol optimized in ESI+ (Figure A3) and thus ionization source settings in the quantitation method were chosen as 290 °C drying gas temperature, 20 L/min drying gas flow rate, 37 psi nebulizer pressure, 400 °C sheath gas temperature, 12 L/min sheath gas flow rate. For ESI+ 6000 V capillary voltage, 2000 V nozzle voltage, high-pressure funnel voltage 110 V, and low-pressure funnel voltage 110 V, and for ESI− 3400 V capillary voltage, 0 V nozzle voltage, 129 V high-pressure funnel voltage, and 121 V low-pressure funnel voltage were selected.

##### Full Factorial Design

**Figure 4:**
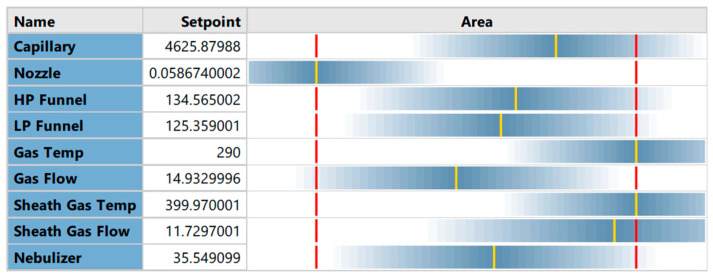
Full factorial design prediction for ion source settings to achieve highest peak area. The full factorial design was performed as CCF quadratic model. Yellow lines show the predicted optimum. Red lines show maximum and minimum of the factor’s range. Gradient of blue color around the yellow lines indicates the predicted decrease of peak area by changing the variable in that region. Unit is V for nozzle, capillary, high-pressure and low-pressure funnel (HP and LP funnel), °C for drying gas temperature (Gas Temp) and sheath gas temperature (Sheath Gas Temp), L/min for sheath gas and drying gas (Gas Flow) flow rate, and psi for nebulizer pressure.

The full factorial design was additionally performed to better investigate the ion source parameters. A prediction for the settings of the source parameters targeting the maximization of the peak area for the quantifier of salbutamol-4′-*O*-sulfate (*m*/*z* = 318.0 → 218.1) was generated (Figure 4).

**Figure 5:**
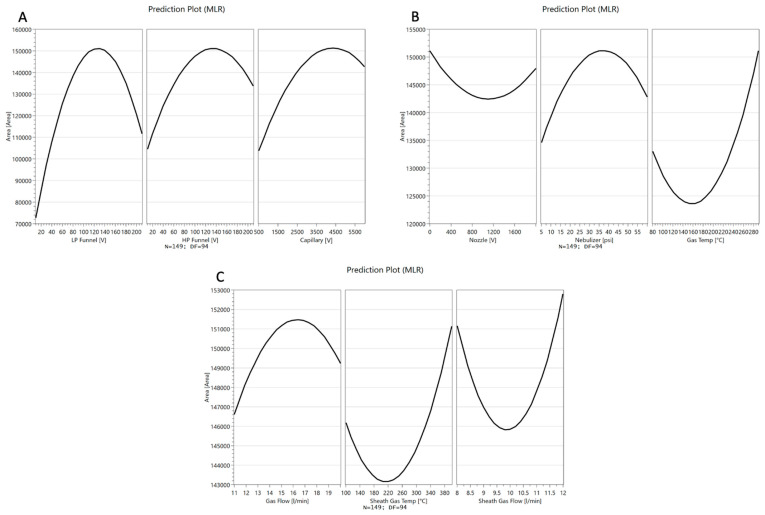
Predicted changes of the peak area for the salbutamol-4′-*O*-sulfate quantifier (*m*/*z* = 318.0 → 218.1) by changing the setpoint of a single variable. The change in the response is shown for adjusting (**A**) low-pressure funnel voltage (LP Funnel), high-pressure funnel voltage (HP funnel), capillary voltage (Capillary), (**B**) nozzle voltage (Nozzle), nebulizer pressure (Nebulizer), drying gas temperature (Gas Temp) and (**C**) drying gas flow (Gas Flow), sheath gas temperature (Sheath Gas Temp) and sheath gas flow (Sheath Gas Flow).

The predicted changes of the peak area of the quantifier of salbutamol-4′-*O*-sulfate (*m*/*z* = 318.0 → 218.1) for each factor, while keeping the other eight factors at the optimized setpoints, were described in parabolas (Figure 5). For low-pressure funnel voltage, high-pressure funnel voltage, capillary voltage, nebulizer pressure, and drying gas flow rate, parabolas show the maximum in the upper midfield of the investigated ranges. Changes in the peak area by altering the nozzle voltage, drying gas temperature sheath gas temperature, and sheath gas flow were described by a parabola opening upwards, and the optimal conditions for ionization were at the end or beginning of the factor ranges. Sheath gas flow rate and temperature as well as drying gas temperature were found to be optimal at the maximum possible values. Furthermore, the nozzle voltage contributes best to the ionization when adjusted to zero.

#### 2.1.3. Peak Identification in Urine Samples through Qualifier-Quantifier Ratios

**Figure 6:**
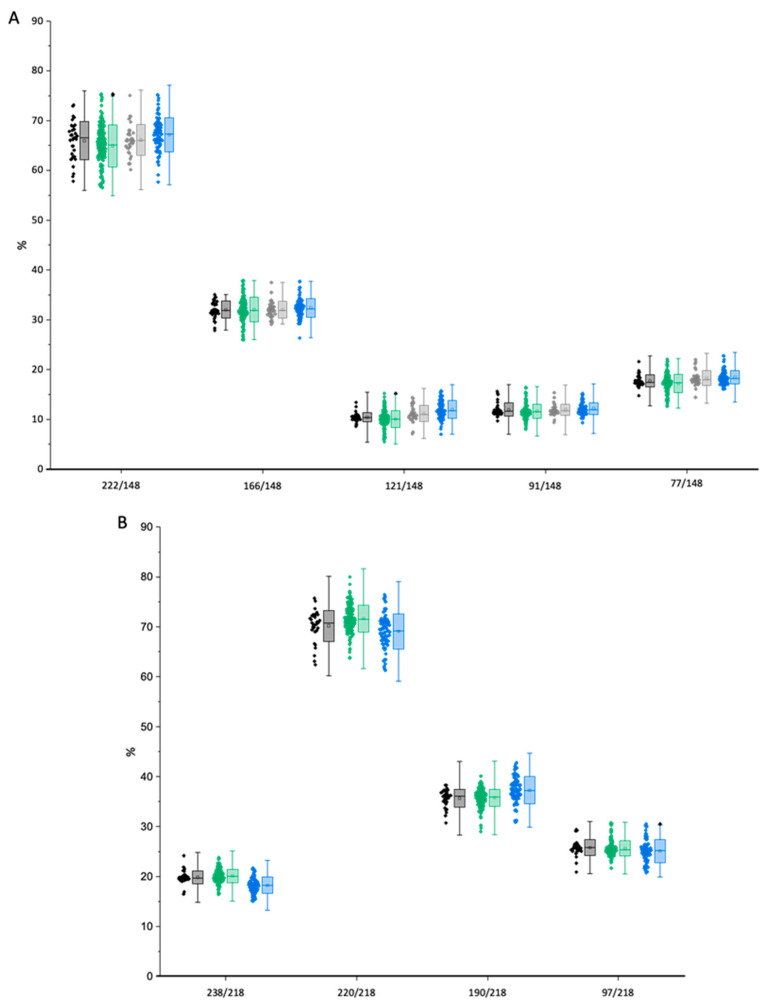
Qualifier-quantifier ratios of (**A**) salbutamol and (**B**) salbutamol-4′-*O*-sulfate. Black refers to calibrants of (*R*)-enantiomer, green to urine samples of (*R*)-enantiomer, grey to calibrants of (*S*)-enantiomer, blue to sample of (*S*)-enantiomer. Box shows standard deviation; whisker shows tolerance for qualifier-quantifier ratios (10% absolute > 50–100%, 10% relative for ratios > 25–50%, and 5% absolute for ratios < 25%). Solid line in box is median; empty square is mean value. Black rhombs highlight outliers.

For identification of the analytes in urine, qualifier-quantifier peak area ratios were calculated from the data of the matrix-assisted calibration. The tolerance for the ratios was calculated according to WADA regulations for identification in tandem mass spectrometry and is shown as whisker in Figure 6. The median values of the calibration were used as reference for the calculation of the tolerance. No outliers were found in the calibration samples and urine samples of (*S*)-salbutamol and (*R*)-salbutamol-4′-*O*-sulfate. For (*R*)-salbutamol, two outliers were found for the qualifier-quantifier-ratio of 148 to 222 (Figure 6A) in two different samples with concentrations of 1.96 ng/mL and 21.66 ng/mL, respectively. One outlier was found for 148 to 121 in a sample with a concentration of 3.76 ng/mL. One additional outlier was found for (*S*)-salbutamol-4′-*O*-sulfate (Figure 6B) at a concentration of 2.98 ng/mL. All outliers were found in urine samples and were related to trace-level concentrations.

### 2.2. Chiral Analysis of Salbutamol-4′-O-sulfate Reference

**Figure 7:**
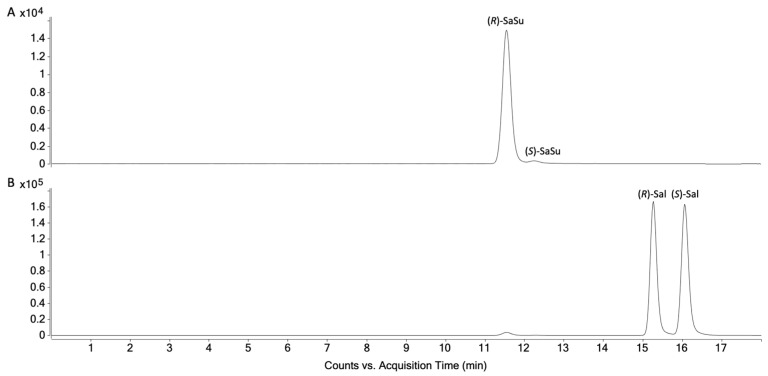
Chromatogram of highest calibration level for (**A**) salbutamol-4′-*O*-sulfate and (**B**) salbutamol.

The biosynthesized and characterized salbutamol-4′-*O*-sulfate reference previously described by Jendretzki and Harps et al. [14] was analyzed with the developed chiral LC-MS/MS method to determine the enantiomeric purity in the reference. The reference was found to contain almost exclusively (*R*)-salbutamol-4′-*O*-sulfate (97.5%) (Figure 7A). 

### 2.3. Excretion Rates of (R)-/(S)-Salbutamol and (R)-/(S)-Salbutamol-4′-O-sulfate

**Figure 8:**
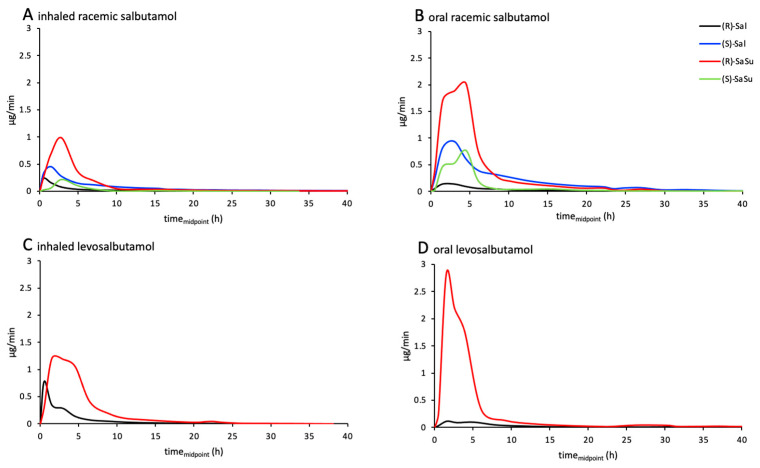
Excretion rates of (*R*)-salbutamol (black lines), (*S*)-salbutamol (blue lines), (*R*)-salbutamol-4′-*O*-sulfate (red lines), and (*S*)-salbutamol-4′-*O*-sulfate (green lines) after (**A**) 600 µg inhaled (SAI) and (**B**) 2 mg peroral (SAP) administration of racemic salbutamol or (**C**) 630 µg inhaled (LSAI) and (**D**) 1 mg peroral (LSAP) administration of levosalbutamol.

Excretion rates of both enantiomers of salbutamol and salbutamol-4′-*O*-sulfate were calculated for both inhaled and oral administration of racemic salbutamol as well as for levosalbutamol. For all administrations, the excretion rates of (*R*)- as well as (*S*)-salbutamol did not exceed 1 µg/min (Figure 8). When administered orally, the maximum excretion rate of (*R*)-salbutamol was lower than the rate for inhaled administration. In contrast, for (*S*)-salbutamol, the maximum excretion rate was higher after oral administration than after inhalation. After racemic salbutamol was administered, excretion rates of (*R*)-salbutamol were lower than for (*S*)-salbutamol.

**Figure A4:**
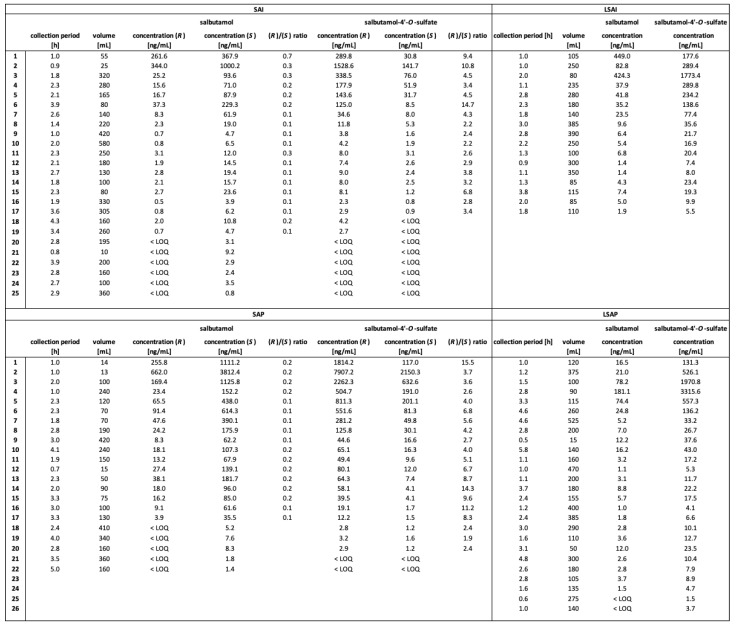
Collection periods, excreted volumes, measured concentrations, and ratio of (*R*)-enantiomer to (*S*)-enantiomer for all trials. SAI—600 µg inhaled racemic salbutamol, SAP—2 mg oral racemic salbutamol, LSAI—630 µg inhaled levosalbutamol, LSAP—1 mg oral levosalbutamol.

For (*R*)- and (*S*)-salbutamol-4′-*O*-sulfate (Figure 8), an opposite correlation was observed. The excretion rates of (*R*)-salbutamol-4′-*O*-sulfate were higher after oral administration than after inhalation, and for the (*S*)-sulfoconjugate, lower excretion rates appeared than for (*R*)-sulfate. Since levosalbutamol only contains the (*R*)-enantiomer, no excretion of (*S*)-salbutamol or (*S*)-salbutamol-4′-*O*-sulfate above the limit of quantification was observed. All measured concentrations as well as excreted volumes of the trials are shown in Figure A4.

### 2.4. Enantiomerical Ratios in Urine

**Figure 9:**
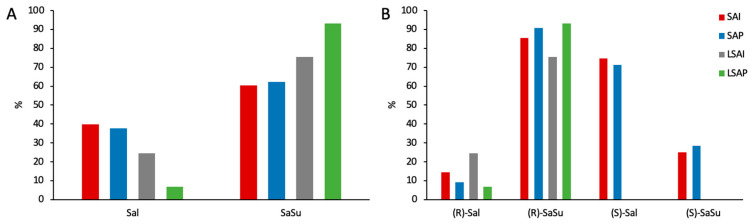
Proportion of recovered amounts in urine found as parent compound or salbutamol-4′-*O*-sulfate (**A**) and proportion of recovered amounts of the enantiomers found as parent compound or sulfate-metabolite (**B**). Red: SAI–600 µg inhaled racemic salbutamol, blue: SAP—2 mg oral racemic salbutamol, grey: LSAI—630 µg inhaled levosalbutamol, green: LSAP—1 mg oral levosalbutamol.

The proportion of parent compound and salbutamol-4′-*O*-sulfate of the recovered amount of the dose in urine was calculated for both enantiomers together (Figure 9A) and separately for the (*R*)- and (*S*)-enantiomers (Figure 9B). The proportion of parent compound excreted was higher after administration of racemic salbutamol than for enantiopure levosalbutamol. Furthermore, the proportion of the sulfate-metabolite was higher after levosalbutamol administration (Figure 9A). When comparing (*R*)- and (*S*)-salbutamol and their metabolites, it was observed that the proportion of excreted (*R*)-parent compound was 5–8 times lower than that of the (*S*)-parent compound and the proportion of (*S*)-sulfate 3–3.5 times lower than that of the (*R*)-sulfate (Figure 9B). The ratios of (*R*)/(*S*) for excreted salbutamol as well as for salbutamol-4′-*O*-sulfate for each sample are shown in Figure A4.

**Figure 10:**
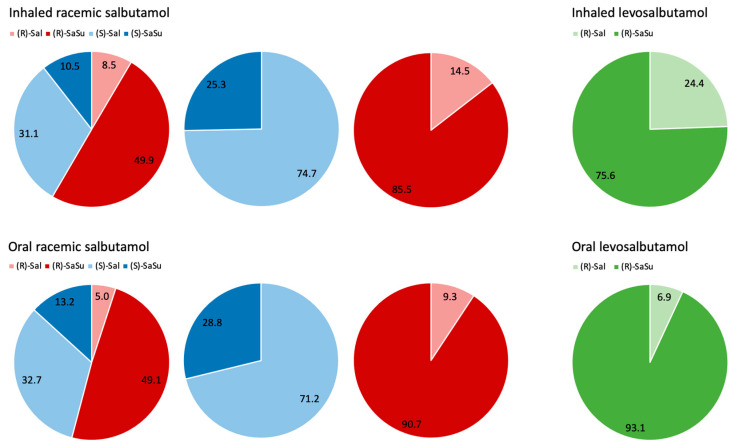
Proportion of (*R*)- and (*S*)-salbutamol and -salbutamol-4′-*O*-sulfate in % of cumulative excreted amount after administration of 600 µg inhaled racemic salbutamol, 2 mg oral racemic salbutamol (red–blue figures), 630 µg inhaled levosalbutamol, 1 mg oral levosalbutamol (green figures). Blue figures show proportion of (*S)*-salbutamol that was sulfonated or excreted as parent compound related to the cumulative excreted amount of the (*S*)-enantiomer. Likewise, red figures show proportion of (*R*)-salbutamol and (*R*)-metabolite related to the cumulative excreted amount of (*R*)-enantiomer.

After administration of racemic salbutamol, more than half of the total urinary excreted amount was recovered as (*R*)-parent compound or its metabolite (Figure 10). Regarding the amount of excreted (*R*)-enantiomer, 85% consisted of (*R*)-salbutamol-4′-*O*-sulfate, while (*S*)-salbutamol was mainly excreted as parent compound after inhalation (SAI: 75%) as well as after oral administration (SAP: 71%). The proportion of sulfate metabolite found in the urine was slightly higher after oral administration for both enantiomers.

When enantiopure levosalbutamol (LSAI, LSAP) was administered, 75% and 93% of the excreted amount, respectively, were detected as the sulfate metabolite, with a higher metabolite proportion after oral application.

Comparing the proportion of metabolized (*R*)-salbutamol after peroral administrations of racemic salbutamol and levosalbutamol, similar proportions of 91% and 93% were found, respectively. After inhaled administration, the proportion of excreted sulfate metabolite was higher after racemic salbutamol than after levosalbutamol.

### 2.5. In-Silico Analysis of the Binding Mode of Salbutamol Enantiomers in SULT1A3

**Figure 11:**
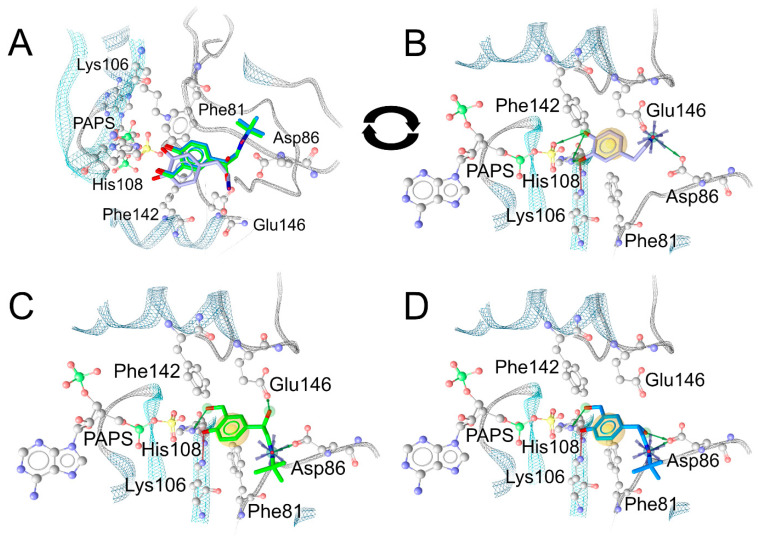
(**A**) Superposition of the co-crystallized conformation of dopamine (violet) with the modeled binding modes of (*R*)- (green) and (*S*)-salbutamol (blue) in their common SULT1A3 binding pocket. For better illustration of the interactions between each individual ligand and the protein residues, (**B**–**D**) depict the pocket after rotation by 180°. (**B**) Binding mode of dopamine. (**C**) Binding mode of (*R*)-salbutamol. (**D**) Binding mode of (*S*)-salbutamol. Violet stars indicate positively charged position, green arrows indicate hydrogen bond donors, red arrows indicate hydrogen bond acceptors, and yellow spheres indicate lipophilic contacts. Black arrows illustrate the rotation of the model comparing (**A**) and (**B**).

To further substantiate the results of salbutamol sulfonation, the binding modes of salbutamol enantiomers in the binding pocket of SULT1A3 with bound PAPS were analyzed in silico. The crystal structure with bound endogenous substrate dopamine and adenosine-3′,5′-diphosphate (PAP) (PDB code: 2A3R [22]) was used as template. Molecular docking was performed after removal of dopamine from the crystallized complex and addition of the sulfate group to transform PAP into PAPS. Plausible binding conformations of the salbutamol stereoisomers in the binding site were picked based on the degree of alignment of the 4-hydroxy group to be sulfonated with the 3-hydroxy group of dopamine as well as their ability to occupy the interaction pattern defined by dopamine. The salbutamol isomers occupy the same binding site as dopamine. Both (*R*)- (Figure 11A, green) and (*S*)-salbutamol (Figure 11A, blue) show good alignment of their aromatic rings with those of the endogenous ligand dopamine (Figure 11A, violet), all of which uniformly engage in lipophilic contacts with Phe81 and Phe142 (Figure 11B–D). The 4-hydroxy group of dopamine forms hydrogen bonds with both an oxygen atom of the PAPS sulfate moiety (Figure 11B) and the ε-nitrogen atom of His108, whereas its 3-hydroxy group is hydrogen-bonded to His108 ε-nitrogen and Lys106 ζ-nitrogen. The 4-hydroxy groups of both salbutamol enantiomers are shown to take on a position close to the 3-hydroxy group of dopamine (Figure 11A), forming hydrogen bonds to the ζ-nitrogen atom of Lys106 (Figure 11C,D). The oxygen atoms of the 3-hydroxy group of dopamine and of the 4-hydroxy groups of (*R*)- and (*S*)-salbutamol are located at distances of 2.8, 3.1, and 3.2 Å to the His108 ε-nitrogen atom, respectively, according to our model. Thus, the hydrogen atoms of the salbutamol phenol groups are well within hydrogen bonding distance. The oxygen atoms of the 3-hydroxy group of dopamine and the 4-hydroxy groups of (*R*)- and (*S*)-salbutamol are 2.8, 3.0, and 3.0 Å, respectively, away from the sulfate group’s sulfur atom with plausible orientation for subsequent sulfonation. The 4-hydroxymethyl groups of both salbutamol enantiomers are aligned with the 3-hydroxy group of dopamine and capable of forming hydrogen bonds with the ε-nitrogen atom of the catalytic His108. In addition, the aliphatic hydroxy group of (*R*)-salbutamol acts as a hydrogen bond donor to one of the ε-oxygen atoms of Glu146. For steric reasons, no such conformation could be obtained for (*S*)-salbutamol, whose hydroxy group points towards Asp86.

## 3. Discussion

### 3.1. Method Development

The chromatographic evaluation led to the optimized separation method utilizing a teicoplanin-based column. The vancomycin-based stationary phase was also found highly suitable for the simultaneous chiral separation of (*R*)-/(*S*)-salbutamol and (*R*)-/(*S*)-salbutamol-4′-*O*-sulfate, whereas the derivatized cyclofructan-based and the hydroxypropylated-β-dextrin-based columns were excluded due to insufficient selectivity after screening. Furthermore, ACN and MeOH with 10 mM AmF on the teicoplanin-based column led to good chiral resolution of the analytes. Adding water to the solvents to ensure elution of all polar matrix ingredients from the urine samples was tested, but water crucially lowered the resolution of the enantiomers. Although adding 50 % (*v*/*v*) ACN to the methanol (Figure 1) led to good enantiomerically selective separation of the analytes, the solvating power for urinary matrix components such as salts would be decreased. Notably, while the ratios of the peaks of (*R*)- and (*S*)-salbutamol-4′-*O*-sulfate were inversed in Figure 1C despite the injection of the same sample, the salbutamol signals at the same retention time as salbutamol-4′-*O*-sulfate caused by in source fragmentation showed the same ratio as the other chromatograms in Figure 1. One explanation might be a strong matrix effect for the (*R*)-salbutamol-4′-*O*-sulfate signal. The use of another mobile phase composition, here also including acetonitrile, might have altered the retention time of matrix components and thus led to coelution with the analyte. This matrix effect did not lessen or impact the underlying salbutamol signal arising from in source fragmentation. However, the signal intensity for salbutamol-4′-*O*-sulfate was much lower when using acetonitrile.

Thus, methanol only was selected as most suitable mobile phase. The optimization of the peak resolution in terms of chiral separation was focused on high resolution to account for extended peak width in isocratic elution mode caused by high concentrations of the analytes. Thus, the separation was performed on a 150 mm teicoplanin-based column run with MeOH and 20 mM AmF.

**Figure 12:**
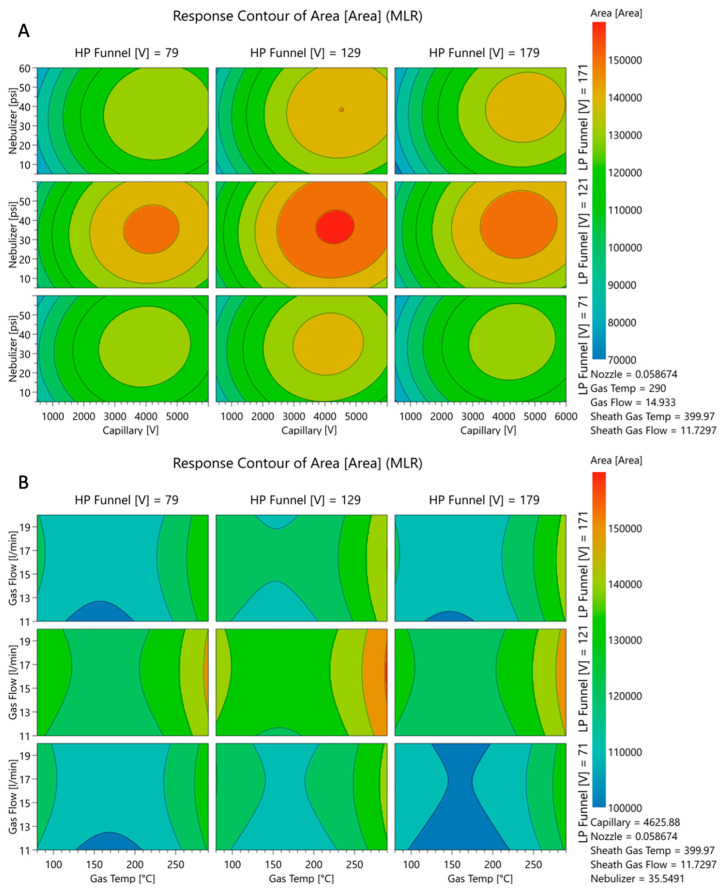
Response 4D shows predicted quantity of response with red as highest and blue as lowest numbers. The response is the peak area of the quantifier of salbutamol-4’-*O*-sulfate as surrogate for ionization efficiency. Values for high-pressure funnel voltage and low-pressure funnel voltage are set to the final applied values 129 V (±50 V) and 121 V (±50V), respectively. In (**A**), the x-axes show capillary voltage and the y-axes show nebulizer pressure. In (**B**), the x-axes show drying gas temperature and y-axes shows drying gas flow rate. On the lower right in (**A**,**B**), the constant variables are shown, which were set to the predicted optimum of the full factorial experiment design. An assessment of the final applied method within the generated knowledge space of the full factorial experiment design was performed.

The MSSD for the optimization of the ion source settings led to a well-optimized ionization setup. The elimination of variables in the method development did not account for the complexity of the ionization. Furthermore, the optimization had to be focused on only one analyte, since the second- and third-step experiments were generated based on the results of the previous step. Referring to the step-by-step optimization of the ionization for the quantifier of salbutamol-4′-*O*-sulfate, the created knowledge space for the method did not allow for evaluation of the results. Only the effects of altering the capillary voltage, low-pressure funnel voltage, and high-pressure funnel voltage were finally described by the quadratic model. A better understanding of the changes in peak areas by altering the setpoints for the variables was achieved by running a full factorial experimental design including all nine factors equally. Figure 12 visualizes the prediction of the peak area—and therefore the ionization efficiency—based on the quadratic model of the full factorial design (FFD). The generated knowledge space around the deviating settings which were used in the final HPLC-MS/MS method for high-pressure funnel, low-pressure funnel, capillary, nebulizer, and drying gas flow are shown. The constant variables in this visualization were set to the predicted optima of the full factorial model (right left corner). Low- and high-pressure funnel were 121 V and 129 V, respectively, in the final HPLC-MS/MS method and are indicated in the third and fourth axes in Figure 12, respectively. Additionally, the predicted effects of capillary voltage and nebulizer pressure (Figure 12A) and of drying gas flow and drying gas temperature (Figure 12B) are shown. When the funnel setting was changed from 50 V to higher or lower values, the red areas were not reachable at all. The crucial impact of the funnel settings was elucidated by both of the prediction models, and the predicted values were only 5 V and 4 V different for low- and high-pressure funnel voltages, respectively. The applied 3400 V for capillary voltage was still in the red area although the FFD predicted capillary voltage was 4626 V (Figure 12A). The nebulizer pressure optimum was predicted via FFD as 35.5 psi and was set after MSSD experiments to 37 psi and thus was still in the red area of the design space. A bigger difference was seen for the drying gas flow rate of 11.7 L/min predicted via FFD and the actual applied drying gas flow rate of 20 L/min in the HPLC-MS/MS method. The red area which indicates optimal condition is only visible as small stripe in Figure 12B. The applied drying gas flow rate would be slightly out of the red area. Only minor differences were seen in the sheath gas flow rate setting compared to the FFD prediction. In conclusion, the applied method systematically developed with the multiple-step screening design was re-evaluated with the more comprehensive design space created with the full factorial design and was classified as highly suitable.

Within the MSSD, an approach was created that optimized the analyte without any matrix, and the predicted values were verified by injecting the spiked urine samples. The results (Figure A3; SaSu− NM; no matrix) indicate the superiority of a matrix-assisted development as it was performed in this study. In another approach, narrower ranges for the investigated ion source variables were applied to the MSSR (Figure A3; SaSu− NR; narrow ranges) leading to a less powerful prediction in which the curvature was not detected and the adjustment from linear to quadratic model was not observed.

### 3.2. Stereoselectivity of Salbutamol Sulfonation

The chiral analysis of the biosynthesized salbutamol-4′-*O*-sulfate reference has shown that the genetically modified fission yeast cells, recombinantly expressing human SULT1A3, have almost exclusively produced (*R*)-salbutamol-4′-*O*-sulfate, supporting the previously reported preference of SULT1A3 towards the (*R*)-enantiomer [9,12].

Similar observations were made in the urine analysis after oral and inhaled administration of racemic salbutamol. (*R*)-salbutamol was more extensively sulfonated than (*S*)-salbutamol and therefore excretion rates and proportions of the excreted amount were lower for the (*R*)-parent compound, concomitantly higher for (*R*)-sulfate and vice versa for (*S*)-salbutamol. When racemic salbutamol was administered, not only was (*R*)-sulfate formed but also, to a lesser extent, (*S*)-salbutamol-4′-*O*-sulfate. Under the biosynthesis conditions using enzyme bags containing SULT1A3, almost exclusively (*R*)-salbutamol-4′-*O*-sulfate was formed. Therefore, the involvement of other SULTs present in the human body besides SULT1A3 in the sulfonation of the (*S*)-enantiomer or the biotransformation by SULT1A3 under different reaction conditions or at different sulfonation rates remains to be investigated.

Comparing the excretion rates of (*R)*-salbutamol after application of racemic salbutamol or levosalbutamol, the excretion rate maximum for (*R*)-salbutamol was higher after enantiopure than after racemic administration. Likewise, the excretion rate maximum of the (*R*)-sulfoconjugate was higher after levosalbutamol application. The higher excretion rate of (*R*)-salbutamol parent compound may be explained by a higher dose of (*R*)-salbutamol in the enantiopure trial (630 µg levosalbutamol vs. 300 µg levosalbutamol in racemic formulation). Referring to routes of administration, it was observed that the proportion of metabolized salbutamol was higher after oral than after inhaled administration. This correlation may be explained by the higher abundance of SULT1A3 in the jejunum than in the lung [8,23] and the higher and complete dose being swallowed compared to lower inhaled and only partly swallowed doses.

In order to find a rational explanation for the predominant sulfonation of (*R*)-salbutamol compared to (*S*)-salbutamol when providing the racemic drug to recombinant human SULT1A3 (expressed in S. pombe), the binding modes of both salbutamol enantiomers in the binding pocket of SULT1A3 with bound PAPS were analyzed. The crystal structure with bound dopamine and adenosine-3′,5′-diphosphate (PAP) (PDB code: 2A3R [22]) was used as a starting point for this structural analysis since dopamine and salbutamol share the 4-(2-aminoethyl)phenol moiety and dopamine is known as endogenous substrate of SULT1A3. While dopamine is equipped with an additional hydroxy group at C2 of the phenyl ring, the salbutamol isomers feature a hydroxymethyl group in position 2. Additionally, the aminoethyl substituent of salbutamol shows an additional hydroxy group at the benzylic position contrary to dopamine and a secondary nitrogen atom decorated with a t-butyl rest takes the place of the terminal primary amine of dopamine. Molecular docking has shown that both salbutamol enantiomers occupy the same binding site as dopamine and show good alignment of their aromatic rings with dopamine and therefore the same lipophilic interaction with Phe81 and Phe142. Dopamine, while equipped with two phenolic hydroxy groups, predominantly circulates as dopamine-3-*O*-sulfate [24,25]. There is significant evidence linking this relative overabundance to preferred sulfonation at the 3-hydroxy position [25]. While the 4-hydroxy group of dopamine aligns with PAPS and His108, the 3-hydroxy group binds to His108 and Lys106. Both Lys106 and His108 play an essential role in facilitating sulfate transfer [26]. Furthermore, it is assumed that His108 acts as catalytic base initiating the catalytic reaction by deprotonating the substrate’s hydroxy group [26]. The 4-hydroxy groups of (*R*)- and (*S*)-salbutamol are positioned similarly to the 3-hydroxy group of dopamine and form hydrogen bonds with the catalytic His108, allowing for sulfonation in this position. For steric reasons, in contrast to (*R*)-salbutamol, the (*S)*-enantiomer does not form a hydrogen bond Glu146. The importance of this glutamate residue for SULT1A3 substrate specificity and catalytic activity has been outlined previously [22,26,27,28]. As this is the most striking difference in the interaction patterns of (*R*)- and (*S*)-salbutamol, we surmise that the hydrogen bond to Glu146 formed by (*R*)-salbutamol contributes to increased binding affinity, consequently increased sulfonation, and accelerated metabolism of (*R*)-salbutamol compared to (*S*)-salbutamol. The nitrogen-containing substituent is trapped between Asp86 and Glu146 in the case of dopamine such that the protonated amine forms ionic interactions with the carboxylate groups of both Glu146 and Asp86. In contrast, the amine-substituents of the two salbutamol enantiomers are positioned in a rotated manner compared to dopamine in our model resulting in charge interactions to Asp86 only. This might be due to the fact that the alcoholic hydroxy group is not capable of forming any hydrogen bonds in our model if the amine-containing substituent is trapped between the two above-mentioned carboxylate moieties. This confirms our observation of the importance of hydrogen bonding by the salbutamol side chain hydroxy group.

## 4. Materials and Methods

### 4.1. Chemicals and Reagents

Salbutamol hemisulfate (>98.0%) and formic acid (for LC-MS) were obtained from TCI Europe (Zwijndrecht, Belgium). Levalbuterol hydrochloride (>98%) and salbutamol-(t-butyl-*d*_9_)-acetate were obtained from Sigma Aldrich (Taufkirchen, Germany). Methanol (MeOH, LC-MS grade) was purchased from Thermo Fisher Scientific (Hennigsdorf, Germany). Ammonium formate (HCOONH_4_, LC-MS grade) was from VWR Chemicals (Darmstadt, Germany). Acetonitrile (ACN, LC-MS grade) was purchased from Honeywell International Inc. (Charlotte, NC, USA). Ultrapure water was prepared with a Milli-Q water purification system LaboStar 2-DI/UV from SG Wasseraufbereitung und Regenerierstation GmbH (Barsbüttel, Germany) equipped with an LC-Pak Polisher and a 0.22-μm membrane point-of-use cartridge (Millipak^®^, Th Geyer, Berlin, Germany). SalbuHEXAL^®^ N was obtained from Hexal AG (Holzkirchen, Germany) and Cyclocaps^®^ Salbutamol from PB Pharma GmbH (Meerbusch, Germany). Xopenex HFA was purchased from Sunovion Pharmaceuticals Inc. (Marlborough, MA, USA) and SALBU-BRONCH^®^ Elixir 1 mg/mL from Infectopharm Arzneimittel und Consilium GmbH (Heppenheim, Germany).

### 4.2. Method Development Chiral Column Chromatography

#### 4.2.1. Chiral Column and Mobile Phase Screening

An initial screening was performed by evaluating four different chiral phases, namely teicoplanin (Agilent InfinityLab Poroshell 120 Chiral-T), vancomycin (Agilent InfinityLab Poroshell 120 Chiral-V), hydroxypropylated-β-cyclodextrin (Agilent InfinityLab Poroshell 120 Chiral-CD), and derivatized cyclofructan (Agilent InfinityLab Poroshell 120 Chiral-CF). The dimensions of the chiral columns were 4.6 mm in diameter and 100 mm length, and they possessed 2.7 µm core-shell particles. Each column was tested with several mobile phase compositions based on methanol (MeOH), acetonitrile (ACN), ultra-pure water (H_2_O), ammonium formate (AmF), and formic acid (FA) (Table 1). The flowrate was set to 0.2 mL/min in isocratic elution mode and the column temperature to 35 °C.

#### 4.2.2. Optimization

Based on the screening results, a teicoplanin-based chiral phase was chosen and used as a 150 mm-long column with a 4.6 mm internal diameter and 2.7 µm core-shell particles for the further optimization. Further optimization included experiments on gradient elution and isocratic elution with flow rates of 0.2 mL/min and 0.4 mL/min applying MeOH, H_2_O, ACN, and AmF as mobile phase. Final fine-tuning implemented testing of AmF in MeOH from 0 to 50 mM stepwise (5 mM) in isocratic elution mode, column temperature from 20 °C to 50 °C in 5 °C steps with 20 mM in MeOH as mobile phase, and flow rates from 0.25 mL/min to 0.5 mL/min.

### 4.3. Optimization of Electrospray Ionization Parameters Ion Source Optimization

Quantitative analysis was conducted on a tandem mass spectrometer (Agilent QQQ 6495C; Agilent Technologies Inc., Santa Clara, CA, USA) equipped with an electrospray ion source with iFunnel technology, which was run simultaneously in negative and positive mode (ESI−, ESI+). The variables which were set up and optimized to enhance ionization efficiency or transfer to the quadrupoles were the flow and the temperature of the drying gas, the pressure of the nebulizer, the sheath gas flow and temperature, the voltage for the capillary and the nozzle, and the voltage for the high-pressure and the low-pressure funnel. The latter four factors can be set up independently for ESI− and ESI+.

Two different experimental designs were created and performed. A multilevel screening design using MiniTab^®^ Statistical Software 21.1.0 (Minitab GmbH, Munich, Germany) and a full factorial design (CCF, quadratic model, 9 factors, 149 runs) using Modde^®^ Pro 13 (Sartorius Data Analytics, Sartorius AG, Göttingen, Germany) were performed. The multistep screening design identified up to five statistically significant factors in the first step (e.g., 9 factors; folded; 24 runs), generated a linear model in the second step (e.g., 4 factors; 19 runs), and adjusted the linear model to a quadratic model in the third step (e.g., 11 extra runs). The full factorial design included 149 experiments which were run as replicates. The targeted analytes salbutamol and salbutamol-4′-*O*-sulfate were provided from one spiked urine sample for each design of experiment, and injection to the QQQ was performed after chiral separation with the same method as all other urine samples (4.5.3.). In general, the optimization was focused on salbutamol-4′-*O*-sulfate. In both experimental designs, the maximal range of the investigated parameters which were technically allowed to choose in the method settings of the ionization source were applied to the experimental design (Table 2). The implemented precursor ions and product ions were optimized in terms of collision energy (CE) and were found via Masshunter Optimizer (Agilent Technologies Inc., Santa Clara; CA, USA). Precursor ion for salbutamol ([M + H]^+^) was *m*/*z* = 240.0 and selected product ions were *m*/*z* = 222.1 (CE 8 eV), 166.1 (CE 12 eV), 148.1 (CE 16 eV), 121.1 (CE 25 eV), 91.0 (CE 48 eV), and 77.1 (CE 56 eV). For salbutamol-4′-*O*-sulfate, precursor ions were ([M + H]^+^) *m*/*z* = 320.0 with product ions *m*/*z* = 240.0 (CE 4 eV), 222.0 (CE 16 eV), 166.0 (CE 16 eV), 148.0 (CE 32 eV), 77.0 (CE 80 eV) and ([M − H]^−^) *m*/*z* = 318.0 with product ions *m*/*z* = 238.0 (CE 25 eV), 220.1 (CE 28 eV), 218.1 (CE 32 eV), 189.9 (CE 44 eV), 97.0 (CE 25 eV), 80.0 (CE 25 eV).

### 4.4. Synthesis of Salbutamol-4′-O-sulfate Reference

To quantitate salbutamol-4′-*O*-sulfate in urine samples, the substance was biosynthesized as reference using a biochemical approach from Sun et al. [10,29] using genetically modified fission yeast strain YN20 expressing SULT1A3. The synthesis, characterization, and quantitation were carried out as described previously by Jendretzki and Harps et al. [14].

### 4.5. Proof of Concept—Longitudinal Excretion Study

#### 4.5.1. Study Design

Racemic salbutamol and pure levosalbutamol were administered to one healthy volunteer as previously described by Jendretzki and Harps et al. [14]. Briefly, single doses of 600 μg of racemic salbutamol (SAI, 6 × 100 μg; SalbuHEXAL^®^ N, Hexal AG, Holzkirchen, Germany) or 630 µg levosalbutamol (LSAI, 14 × 45 μg; Xopenex HFA^®^, Sunovion Pharmaceuticals Inc., Marlborough, MA, USA) were applied via inhalation as aerosol. Additionally, oral administrations of 2 mg of racemic salbutamol (SAP, 2 mL as drops; SALBU-BRONCH^®^ Elixir 1 mg/mL, Infectopharm Arzneimittel und Consilium GmbH, Heppenheim, Germany) and 1 mg of levosalbutamol hydrochloride (LSAP, 2 mL of a 0.5 mg/mL levosalbutamol solution) were performed. Administrations were carried out at least one week apart to ensure full washout. Urine was collected pre- and up to 6 days post-administration. All urine samples were collected as they accrued throughout at least the first 48 h after administration. Afterward, morning urines were collected. Excreted volumes and corresponding collection periods were recorded. Aliquots of the urine samples were stored at −20 °C until analysis.

#### 4.5.2. Matrix-Assisted Calibration

Matrix-assisted calibration was performed with the biosynthesized (*R*)-salbutamol-4′-*O*-sulfate reference. Calibration levels in a range from 0.8 ng/mL to 797 ng/mL for (*R*)-salbutamol-4′-*O*-sulfate and 0.62 ng/mL to 622 ng/mL for (*R*)- as well as (*S*)-salbutamol were prepared with analyte-free urine. Since the synthesized reference only yielded (*R*)-salbutamol-4′-*O*-sulfate, it was also used as calibration for (*S*)-salbutamol-4′-*O*-sulfate.

#### 4.5.3. Sample Preparation

Urine samples were thawed at room temperature. Aliquots of 200 µL of urine sample were then diluted with 700 µL methanol and 100 µL methanolic solution of internal standard (final concentration of internal standard 500 ng/mL). Samples were vortexed, cooled at −20 °C for 10 min, and subsequently centrifuged at 14,100 rcf for 5 min. The supernatant was then transferred to glass vials for analysis.

#### 4.5.4. Instruments and Chromatographic Conditions

Urine analysis was carried out via ultrahigh-performance liquid chromatography-tandem mass spectrometry (UHPLC-MS/MS) using a 1290 Infinity II UHPLC-System (Agilent Technologies, Waldbronn, Germany) coupled with a 6495 iFunnel triple quadrupole (QQQ) MS (G6495B; Agilent Technologies, Santa Clara, CA, USA). The final chiral chromatography method was performed utilizing an Agilent InfinityLab Poroshell Chiral-T column (4.6 mm I.D. × 150 mm; 2.7 μm) at a column temperature of 35 °C. Isocratic elution was performed using 20 mM ammonium formate in methanol, and the flow rate was 0.2 mL/min for 13.5 min and was then enhanced to 0.4 mL/min within 0.05 min for 4.45 min.

The tandem mass spectrometer was operated in positive and negative electrospray ionization (ESI+ and ESI−) modes using multiple reaction monitoring (MRM). Ion source parameters were set to 290 °C drying gas temperature, 20 L/min drying gas flow, 37 psi nebulizer, 400 °C sheath gas temperature, and sheath gas flow 12 L/min. In ESI−, capillary voltage was 3400 V, nozzle voltage 0 V, high-pressure funnel 129 V, and low-pressure funnel was 121 V. In ESI+, capillary voltage was 6000 V, nozzle voltage 2000 V, high-pressure funnel 110 V, and low-pressure funnel was 110 V. Precursor ions and product ions for the MRM method are available in Table 3.

### 4.6. Protein Preparation and Molecular Docking

Out of the three SULT1A3 crystal structure available from the Protein Data Bank (PDB) [30], the structure with co-crystalized dopamine (PDB code: 2A3R [22]) was chosen as dopamine and the ligands of interest, the salbutamol isomers, share the same chemical structure with different substituents. The protein structure was prepared using the MOE (Molecular Operating Environment 2022.02; Chemical Computing Group ULC, Montreal, QC, Canada) package in the implemented OPLS-AA force field [31]; co-crystallized water molecules as well as co-crystallized dopamine and the additional protein chain were manually removed. The cofactor adenosine-3′,5′-diphosphate (PAP), representing the depleted manifestation of the cofactor, was manually transformed into PAPS and subsequently energetically minimized; the structure was protonated at 300 K and pH 7 employing Protonate3D in MOE [32]. Ramachandran plot outliers and atom clashes were manually fixed via local energy minimization within the MOE Protein Builder functionality and inbuilt MOE loop modeler tool in the OPLS-AA force field [31]. Molecular docking of (*R)*- and (*S)*-salbutamol into their SULT1A3 binding pocket was conducted using GOLD v. 5.8.1 (Genetic Optimization for Ligand Docking, CCDC Software, Cambridge, UK) [33]. Pyramidal nitrogen atoms were allowed to flip. The coordinates of the sulfur atom of the PAPS cofactor were chosen as center of the docking operation with a sphere of 10 Å. The genetic algorithm (GA) was run 50 times. The algorithm was aimed at obtaining diverse docking conformations with a root mean square difference (RMSD) of at least 1.5 Å, early termination functionality was turned off, and the PLP scoring function was employed. Apart from that, default conditions were kept. The retrieved conformations of (*R*)- and (*S*)-salbutamol were energy-minimized in the MMFF94 forcefield [34] in LigandScout v.4.4.3 (Inte:ligand, Vienna, Austria) [35], and visually inspected in the same. Plausible poses were chosen based on the alignment of their respective 4-hydroxy group with the 3-hydroxy group of the co-crystallized endogenous ligand dopamine and their ability to occupy the same interaction positions with the protein as dopamine.

### 4.7. Data Analysis

The resolution (*R*) of the peaks was calculated with Agilent MassHunter Quantitative Analysis 10.1 (Agilent Technologies, Santa Clara, CA, USA) according to the European Pharmacopoeia with the retention times of the peaks (*t_R_*_1_, *t_R_*_2_) and the peak width at half of the peak height (*w_h_*_1_, *w_h_*_2_) using the following equation:

$$R=\frac{1.18\times (t_{R2}-t_{R1})}{w_{h1}+w_{h2}}$$

Excreted masses (*m*) per collection timeframe for each analyte were calculated as salbutamol equivalent using the measured concentration (*C*) and the recorded excreted volume (*V*). The following equation was used:

m=C×V

The total excreted amount (*m_excreted_*) of each analyte was calculated as sum of the excreted amounts of the analytes per timeframe (*m_analyte(t)_*). Salbutamol-4′-*O*-sulfate was calculated as salbutamol equivalent.

$$m_{excreted}=\sum m_{analyte(t)}$$

All calculations for the proof-of-concept trial were performed in Microsoft^®^ Excel 16.71 (Munich, Germany). Qualifier–quantifier ratios and statistical evaluation was performed in OriginPro^®^ 2019 (Academic) (OriginLab Corporation, Northampton, MA, USA).

## Figures and Tables

**Table 1:** Mobile phases used for the screening of teicoplanin, vancomycin, hydroxypropylated-β-cyclodextrin, and derivatized cyclofructan based chiral columns.

	MeOH	ACN	H_2_O	AmF	FA
1	50%	50%	--	10 mM	--
2	50%	50%	--	10 mM	0.05%
3	100%	--	--	20 mM	--
4	100%	--	--	--	0.1%
5	100%	--	--	5 mM	0.075%
6	100%	--	--	10 mM	0.05%
7	100%	--	--	15 mM	0.025%
8	95%	--	5%	20 mM	--
9	90%	--	10%	20 mM	--
10	80%	--	20%	20 mM	--
11	70%	--	30%	20 mM	--

-- not contained in mobile phase.

**Table 2:** Ranges for the variables optimized in electrospray ionization.

Capillary voltage *	500 V to 6000 V
Nozzle Voltage *	0 V to 2000 V
High-pressure funnel voltage *	10 V to 210 V
Low-pressure funnel voltage *	10 V to 210 V
Drying gas temperature	80 °C to 290 °C
Drying gas flow rate	11 L/min to 20 L/min
Sheath gas temperature	100 °C to 400 °C
Sheath gas flow rate	8 L/min to 12 L/min
Nebulizer pressure	5 psi to 60 psi

* Independently adjustable in ESI− and ESI+ in same source ionization method.

**Table 3:** Precursors, product ions, and collision energies used in multiple reaction monitoring modes.

Compound	Precursor Ion [*m*/*z*]	Product Ion [*m*/*z*]	Collision Energy [eV]
(*R*)-/(*S*)-Salbutamol	[M + H]^+^ = 240.0	222.1	8
		166.1	12
		148.1 *	16
		121.1	25
		91.0	48
		77.1	56
(*R*)-/(*S*)-Salbutamol-4′-*O*-sulfate	[M − H]^–^ = 318.0	238.0	25
		220.0	28
		218.1 *	32
		190.0	44
		97.0	25
*d*_9_-Salbutamol	[M + H]^+^ = 249.0	231.1	8
		166.1	12
		148.1	16
		121.1	25

* Transitions of highest intensity; quantifier.

## Data Availability

Data are available from the authors upon request.
